# Portable Electrochemical Biosensors Based on Microcontrollers for Detection of Viruses: A Review

**DOI:** 10.3390/bios12080666

**Published:** 2022-08-22

**Authors:** Muhammad Afiq Abdul Ghani, Anis Nurashikin Nordin, Munirah Zulhairee, Adibah Che Mohamad Nor, Mohd Shihabuddin Ahmad Noorden, Muhammad Khairul Faisal Muhamad Atan, Rosminazuin Ab Rahim, Zainiharyati Mohd Zain

**Affiliations:** 1 MEMS-VLSI Research Unit, Department of Electrical and Computer Engineering, Engineering Faculty, International Islamic University Malaysia, Kuala Lumpur 53100, Federal Territory of Kuala Lumpur, Malaysia; 2 Electrochemical Material and Sensor (EMaS) Research Group, Faculty of Applied Sciences, Universiti Teknologi MARA, Shah Alam 40450, Selangor, Malaysia; 3 Faculty of Pharmacy, Universiti Teknologi MARA, Puncak Alam Campus, Bandar Puncak Alam 42300, Selangor, Malaysia

**Keywords:** electrochemical biosensors, virus detection, RT-LAMP, potentiostat, electrochemical impedance spectroscopy, voltammetry, amperometry

## Abstract

With the rise of zoonotic diseases in recent years, there is an urgent need for improved and more accessible screening and diagnostic methods to mitigate future outbreaks. The recent COVID-19 pandemic revealed an over-reliance on RT-PCR, a slow, costly and lab-based method for diagnostics. To better manage the pandemic, a high-throughput, rapid point-of-care device is needed for early detection and isolation of patients. Electrochemical biosensors offer a promising solution, as they can be used to perform on-site tests without the need for centralized labs, producing high-throughput and accurate measurements compared to rapid test kits. In this work, we detail important considerations for the use of electrochemical biosensors for the detection of respiratory viruses. Methods of enhancing signal outputs via amplification of the analyte, biorecognition of elements and modification of the transducer are also explained. The use of portable potentiostats and microfluidics chambers that create a miniature lab are also discussed in detail as an alternative to centralized laboratory settings. The state-of-the-art usage of portable potentiostats for detection of viruses is also elaborated and categorized according to detection technique: amperometry, voltammetry and electrochemical impedance spectroscopy. In terms of integration with microfluidics, RT-LAMP is identified as the preferred method for DNA amplification virus detection. RT-LAMP methods have shorter turnaround times compared to RT-PCR and do not require thermal cycling. Current applications of RT-LAMP for virus detection are also elaborated upon.

## 1. Introduction

Rapid modernization and the increase in human population in recent years have created immense pressure on the Earth’s ecosystem, destroying natural habitats and causing biodiversity loss and climate change. Intensified development often encroaches on natural habitats, which leads to multiplied interactions between humans and wildlife, increasing the likelihood of transmission of viruses or diseases and causing outbreaks [1]. The recent COVID-19 pandemic has highlighted the crippling effects of pandemics on human health and the global economy. Since its emergence in early 2020 and up until the end of May 2022, the coronavirus SARS-CoV-2 has caused more than 524 million COVID-19 cases globally, with 6.2 million deaths (~1.2% case fatality rate or CFR) [1,2]. Today, the risk of concurrent pandemics or the onset of future pandemics is inevitable. Zoonotic viruses represent the greatest threat to global health, including not only coronaviruses, such as Middle East Respiratory Syndrome (MERS-CoV) [3], but also hemorrhagic fever viruses, hantaviruses, arenaviruses, arboviruses [4] and zoonotic influenza viruses [5,6,7,8]. The number of casualties of the SARS-CoV-2 virus is high, resulting in at least six million deaths, with multiple variants due to mutations [2]. 

A key factor that can mitigate outbreaks is early detection and isolation. The availability of diverse methods for virus detection, such that screening can be implemented rapidly, efficiently and in locations throughout the world, is crucial. Several methods are available to detect viruses such as COVID-19, such as molecular, antigen and serological methods [3]. During the early days of the COVID-19 pandemic, the world heavily relied on gene sequencing and reverse transcription-polymerase chain reaction (RT-PCR) to diagnose infected patients [1]. Unfortunately, RT-PCR-based tests are slow (3–4 h) and require complex preparation of samples, expensive lab equipment and facilities and trained personnel that are only commonly available in first-world countries [3]. This makes middle- to low-income countries especially vulnerable to pandemics, as they do not have the luxury of high-tech diagnostic laboratories. To mitigate future outbreaks, the provision of simplified yet accurate virus detection methods that allow for high-throughput, large-scale, accurate and inexpensive screening is a top priority [4]. 

To overcome the drawbacks of RT-PCR, researchers have developed alternative virus detection methods, such as the point-of-care testing (POCT) method, to allow for detection of viruses outside of laboratory settings. Among them are (i) a one-step reverse transcription LAMP (loop-mediated isothermal amplification) method that uses nucleic acid detection and can be used without sophisticated equipment [5]; (ii) the CRISPR (clustered regularly interspaced short palindromic repeat) method, which uses a chromatographic strip to detect target nucleic acids; and (iii) colloidal gold- and fluorescence-based immunochromatographic assays [6]. A comparison of competing POCT methods for virus detection, such as LAMP and CRISPR methods, is shown in Table 1. 

LAMP is a powerful isothermal amplification assay that allows for quick and sensitive detection of specific genes for disease diagnosis [7]. LAMP can provide sensitivities of fg or <10 copies of the target nucleic acid. The LAMP methodology involves enzymatic amplification of a desired nucleic acid region using six DNA primers at a constant temperature of 65 °C. *Bst* DNA polymerase (*Bacillus stearothermophilus*) amplifies according to the target DNA or RNA genomic template as its complementary template. LAMP amplification occurs through a series of primer annealing, strand displacement, self-annealing and formation of a loop shape at one end [8]. LAMP amplification results can be verified by agarose gel electrophoresis, colorimetric observation [9] and real-time fluorimetry [10,11]. In areas with limited resources, 65 °C incubation can be achieved using water baths, and detection can be performed by the naked eye. 

Another commonly used method for virus detection is enzyme-linked immunosorbent assay (ELISA). ELISA is generally a lab-based test to detect antibody–viral protein complexes using fluid from patients. This plate-based assay method can detect soluble substances, such as peptides, proteins, antibodies and hormones. For example, neutralization assays provide quantitative information on the ability of patient antibodies to confer protective immunity. ELISA can also detect and quantify human IgG, IgM or total Igs from serum or plasma samples from SARS-CoV-2-positive individuals. Immunodetection methods have emerged as an alternative to SARS-CoV-2 monitoring, owing to their potential to provide rapid testing results; however, antibody response profiles require a certain duration time after infection, and false negatives due to the poor accuracy and low sensitivity of these testing methods can exacerbate the spread of SARS-CoV-2 [12,13]. 

Another popular detection method is clustered regularly interspaced short palindromic repeats (CRISPR). This method involves gene editing. In CRISPR-Cas system-based diagnostic methods, guide RNA or CRISPR RNA (crRNA) are designed to match specific sequences of desired target genes or regions that indicate the presence of the disease. CRISPR RNA (crRNA) guides Cas protein as it forms complexes with the protein to recognize and cleave specific matching or complementary sequences [14]. Cas12 and Cas13 are the two examples of the most commonly used Cas proteins in CRISPR-based diagnostic assay. In cis-cleavage, Cas12 recognizes and cuts the complementary DNA and Cas13 recognizes and cuts on the complementary RNA [15,16]. Then, Cas protein initiates trans-cleavage [17] which occurs at various locations on the protein and involves cutting of the nucleic acid sequences that do not bind to the CRISPR RNA [14]. For most diagnostic applications of the CRISPR-Cas system, a labelled fluorescent reporter is placed such that it will light up when target genes are cut, indicating positive detection of the disease [15,18]. The designed CRISPR/Cas13a biosensor enables preamplification-free detection of ultra-low concentrations of SARS-CoV-2 RNA and on-site and rapid diagnostic testing for COVID-19 [19]. However, CRISPR-based optical detection strategies require a bulky and expensive optical device, which can restrict their applicability in POCT-related contexts. Electrochemical measurement techniques can quantify low amounts of a target gene, owing to their high sensitivity, specificity, simplicity of miniaturization, portability and cost-effectiveness.

**Table 1:** Comparison of existing virus detection methods: LAMP, CRISPR, ELISA and electrochemical methods.

	LAMP	CRISPR	ELISA	qPCR	Electrochemical
Temperature	65 °C	37 °C	Room temperature	3 temperatures: 95 °C, 60 °C and 70 °C	Room temperature
Material to enhance electrochemical signal	Fluorescent dye, active nanoparticles, magnetic beads or redox materials		Viral protein	Viral nucleic acid	Viral particle/nucleic acid
Nucleic acid primer bases	Specific forward and backward internal primers with at least 100 bases in length to match with the virus-targeted gene sequence	CRISPR RNA designed to match a specific sequence of the desired target gene or region in the virus	Antibody and ligand binding	3–4 h Forward and reverse labelled primers	Less than 1 h Hybridization of complementary DNA probe
Advantages	Low cost Specificity Highly effective detection Rapid monitoring strategies	Highly effective detection and rapid monitoring strategies	Specificity through antibody affinity	Specific and highly sensitive signal, shows real-time infection	Low cost, i.e., it can be reprinted and reused. Specificity through full-match hybridization, simplicity of miniaturization
Disadvantages	A heat block is needed for on-site detection, which might be solved by embedding a fluidic channel or mini reactor coupled with a heat regulator	Expensive RNA unstable in nature, setting for enzymatic reaction, Well-trained personnel	Not at the time of infection; time is required for antibody production	A qPCR terminal cycler is needed for on-site detection, setting for enzymatic reaction, Well-trained personnel	Requires a portable detector for on-site application, orientation of the immobilized ssDNA influence the result
References	[8,10,20,21,22]	[14,15,16,17,18]	[12,13]	[3]	[23,24,25]

Another widely used method with considerable potential as a POCT is electrochemical biosensors. Electrochemical biosensors detect chemical interactions and transduce them into electrical signals, allowing for precise target monitoring via miniature devices [26]. An important component of an electrochemical biosensor system is the potentiostat. A potentiostat is an electronic device used to control and measure electrochemical cells [27]. High-precision, lab-grade potentiostats are not portable and are costly. Therefore, it is important for potentiostats to be highly accessible in terms of usage and reach around the world; furthermore, potentiostats need to be portable, with adequate precision to detect specific viruses. Several studies have been conducted to date on portable potentiostats, such as the ABE-Stat [28], SIMstat [29] and Kickstat [30]. However, these devices are still in the early development stage and have not yet been employed for clinical testing and virus detection.

Whereas numerous methods are available to improve the sensitivity and selectivity of sensors, there has not been much discussion with respect to the development of reliable, portable devices for virus screening. In this paper, we intend to review the state of the art of the usage of portable potentiostats as screening of viruses. The remainder of the paper is organized as follows. In Section 2, we explore the detection of viruses using electrochemical biosensors, including the theory of biosensors, as well as important design considerations for biosensors with respect to viruses. In Section 3, we analyze available portable potentiostats and their measuring capabilities. Finally, And we present our conclusions in Section 4. 

## 2. Detection of Viruses Using Electrochemical Biosensors

**Figure 1:**
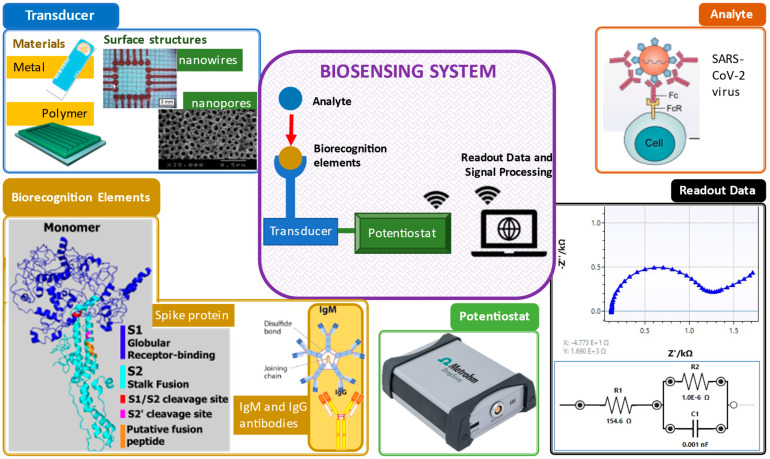
Important elements in a biosensing system. Clockwise from top right: analyte [32], electrochemical impedance spectroscopy readout data, potentiostat [33], biorecognition elements (spike protein [34] and antibodies [35]) and transducers and their surface structures (screen-printed gold electrode [36], polymer electrode [37], nanowire structure [38] and nanopore structure [39]).

An electrochemical biosensor plays three crucial roles: biorecognition, transduction and signal output, as shown in Figure 1 [31]. 

In general, the principle of biosensors involves, first, the identification of biorecognition to the target samples. Next, the transducer or electrodes detect any changes due to the interaction of both the target and biorecognition. Then, the transducer translates the interaction changes to a digital detector. Lastly, the digital output signals are displayed by digital devices, such as smartphones or laptops [40]. The digital output signals can be described as the electrochemical response, including the increase (signal on) and decrease (signal off) of the target concentration elevation by the electrodes measured via either electron transfer or electron transfer resistance [41]. With the existing biorecognition elements mixed with the analyte, existence of virus detected in the sample can be confirmed.

### 2.1. Theory of Electrochemical Biosensors 

**Figure 2:**
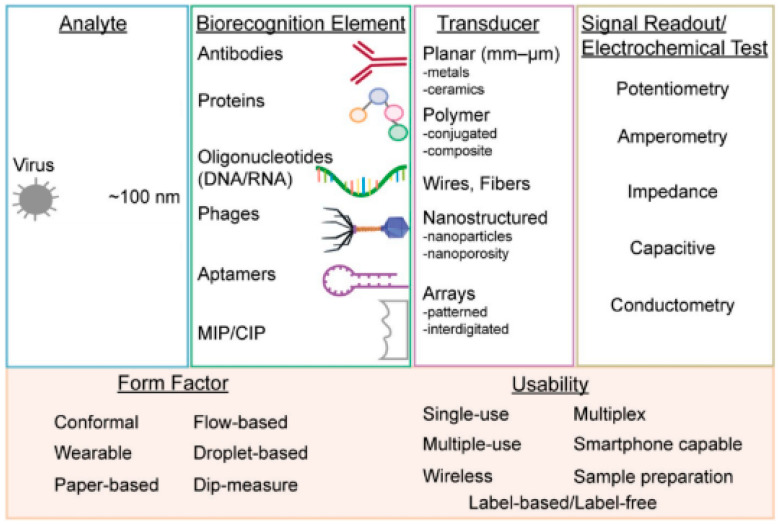
From left to right: virus form factors, types of biorecognition elements, methods to enhance the sensitivity of transducers, various measurement techniques using electrochemical testing and the multi-usability of electrochemical biosensors [26].

Electrochemistry is one of the key measuring methods to view reactions involved in electron transfers [42]. Electrochemical biosensors usually contain biological recognition elements that react selectively with the target analytes. Biological recognition elements are typically enzymes, antibodies or nucleic acid [43] that react to the tested analytes, such as viruses, as shown in Figure 1. Electrochemical biosensors can be divided into two categories: biocatalytic sensors and affinity biosensors. Biocatalytic sensors use enzymes as recognition elements and are commonly used for blood glucose monitoring, whereas affinity sensors use biomolecules, such as antibodies, oligonucleotides or commonly used aptamers, as biorecognition elements [26]. Affinity biosensors typically utilize antibodies or aptamers for virus detection. Figure 2 shows the important components that are necessary for successful virus detection. 

Various electrochemical techniques that can be used for biosensing, such as voltammetry, impedance spectroscopy, amperometry, chronopotentiometry, etc. Voltammetry measures current when different potentials are applied. By performing voltammetry, voltage and current characteristics of a medium can be determined, as well as mechanistic and kinetic details of electron transfer [44]. Cyclic voltammetry (CV) is usually used to investigate the reduction process of molecular species, as well as oxidation processes, making it important to study the chemical reactions that involve electron transfer and can also be used to characterize the stability of electrodes [42]. Electrochemical impedance spectroscopy (EIS) is a technique used to investigate electrical properties of various forms of matter by measuring the current response after applying sinusoidal voltage [45]. According to the current response, the data can be represented in three forms (Fourier transform) in order to decompose the result to assess the magnitude and frequency in the form of a bode plot to measure the frequency response of systems, in addition to a Nyquist plot. The results from the Nyquist plot can determine double-layer capacitance, the ohmic resistance of the electrolyte solution, Warburg impedance and electron transfer resistance. EIS is also a powerful technique that can be used to analyze the electron transfer kinetics and for the detection of molecular interactions. 

These three electrochemical techniques each have their advantages and disadvantages depending on how they are executed. With respect to time taken for execution, amperometry and voltammetry techniques can be applied with a shorter duration than EIS. The execution time usually depends on the scan rate [46], which impacts the time taken for the operation to complete, which can be as short as 10 s or as long as a few minutes. However, electrochemical impedance spectroscopy (EIS) techniques take much longer. Normally, EIS is executed in the frequency range of 0.1Hz to 100KHz. The time taken for EIS to be executed in this range usually depends on the microcontroller performances of the potentiostat. For example, EIS running on μStat-i 400 from MetroOhm with the stated frequency range and 10 readings per decade takes approximately 10-15 min, whereas with the Pico development kit from Palmsens, the same operations takes approximately 27 min. 

Impedance characterization of the electrode–electrolyte interface is important in the field of impedance-based biosensing. EIS is a useful technique, as it captures the phenomena that occur at the electrode interface. Impedance is measured by applying a sinusoidal voltage to the electrochemical cell and measuring the produced current. The mathematical basis of the system can be explained by Equation (1) [47].

*Z* = *E_t_*/*I_t_* (1)

where *Z* is impedance, and both *E_t_* and *I_t_* are potential and current at a specific time instant. A phase shift occurs between the applied voltage and the current due to the capacitive and resistive effects of the electrochemical system [48]. The electrode–electrolyte interface includes a charge transfer resistance and capacitance in combination with the resistance of the solution resistance. This impedance data over a set frequency range can be informative with respect to any ionic interactions or modifications that occur on the surface of the electrode. Equivalent circuit models, such as Warburg circuits, can be used to model these interactions and can be curve-fitted to the measured data. The selected model of the equivalent circuit is deemed acceptable if it has low chi-squared values (χ^2^) [49]. 

In terms of measurement durations, amperometry and voltammetry are far superior to EIS. However, timing performance is not the only indicators that needs to be taken into consideration. Amperometry and voltammetry techniques can be used to detect electron transfer through oxidation/reduction reaction by supplying currents or potentials [50]. EIS has advantages over amperometry in terms of direct antibody–antigen binding detection without applying high potentials to induce a reduction/oxidation reaction [50]. Therefore, all the mentioned advantages and disadvantages should be considered selecting the optimal techniques for detection. 

### 2.2. Important Design Considerations for Detection of Viruses

One important consideration for detection of viruses is that the size of a virus is smaller than that of bacteria [51]. Most viruses have sizes ranging from 10 to 100 nm, compared to bacteria, which are at least 10 times smaller, given that the typical bacterium is approximately 1 µm in size [52]. Because sizes are in the nanometer range, other than sensitivity and selective biorecognition elements, detection of viruses often requires amplification, immobilization of sensing materials and surface passivation to enhance binding reactions [26]. 

#### 2.2.1. Transducing Elements

A key component of a biosensor is the transducer, which can transform chemical interactions on the sensing surface into electrical signals via electrodes. Typically, a three-electrode format is used in electrochemical systems. Electrodes can be fabricated from conducting and semi-conducting materials, such as carbon, which is widely used, as well as gold (Au). Electrode designs can vary in terms of form factors, usability, materials as and fabrication method. Integrated electrodes can be manufactured cost-effectively using techniques such as screen printing and lamination methods. Screen-printed electrodes (SPEs) are commercially manufactured and can produce stable, reliable results, making them ideal as a cost-effective solution for high-throughput screening that requires disposable devices. Polymer electrodes have also been used for virus detection. One example is a graphene–polymer electrode used for detection of dengue virus (DENV) [53]. The use of polymers as electrode elements is associated with various advantages, such as tunable electrical conductivity, biocompatibility and environmental stability. Electrodes are also compatible with multiple ranges of immobilization techniques and biorecognition elements [54,55]. 

**Figure 3:**
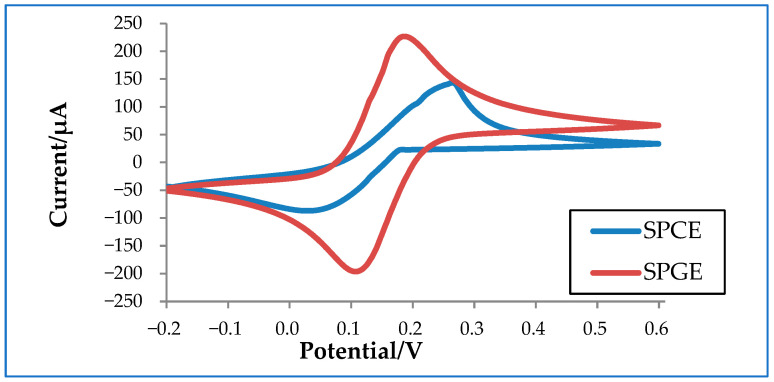
Cyclic voltammetry graph of a commercialized screen-printed carbon electrode (SPCE) and a screen-printed gold electrode (SPGE) in 10 mM ferrocyanide.

In order to better understand how electrode materials impact detection, an experiment was conducted to compare the performance of commercialized carbon and gold electrodes. Carbon has a conductivity of 1.25 × 10^3^ to 2.00 × 10^3^ S/m at 20 °C, whereas gold has a much higher conductivity of 4.11 × 10^7^ S/m at 20 °C. As shown in Figure 3, SPGE shows higher oxidation and reduction peaks compared to SPCE due to its higher conductivity. Despite its superior performance, SPGE is less commonly used compared to SPCE due to its higher associated costs. Thus, a considerable amount of research has been conducted to tackle this issue in an attempt improve the sensitivity of SPCE, mainly by increasing the electrode surface area. The larger the surface area, the higher the sensitivity of the electrode, which is particularly crucial for virus detection [56,57]. 

**Figure 4:**
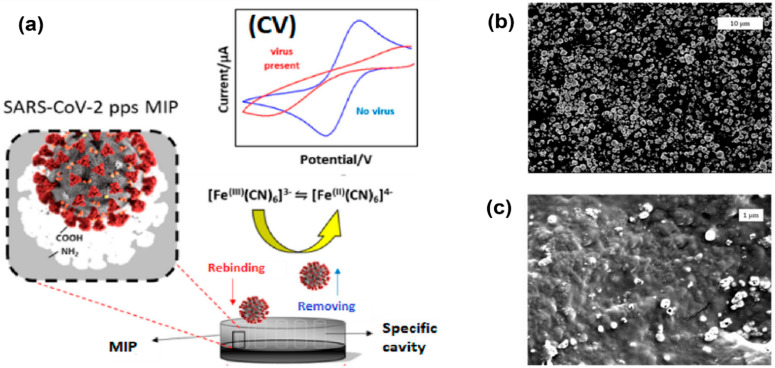
Electropolymerization technique using molecular imprinted polymer (MIP) to enhance the surface area and sensitivity of electrodes. (**a**) Imprinted MIP is electrodeposited on a screen-printed carbon electrode to create enhanced anodic/cathodic currents during cyclic voltammetry measurements. (**b**) Electrode surface before polymerization. (**c**) Electrode surface after polymerization, indicating polymer modification and a change in topography [59].

Higher surface area can be achieved by nanostructure formations on top of the electrodes. Examples of these nanostructures are nanowires and nanopores [58]. Molecular imprinted polymers (MIPs) can also be used to enhance the surface area and result in improved electrode performance, as shown in Figure 4 [37]. In this work, MIPs were electropolymerized on commercial carbon electrodes to produce an enhanced cyclic voltammetry response for detection of SARS-CoV-2 viruses. The peak separation and current values are much higher in electrodes with a nanoporous gold thin film compared to bare gold electrodes. 

**Figure 5:**
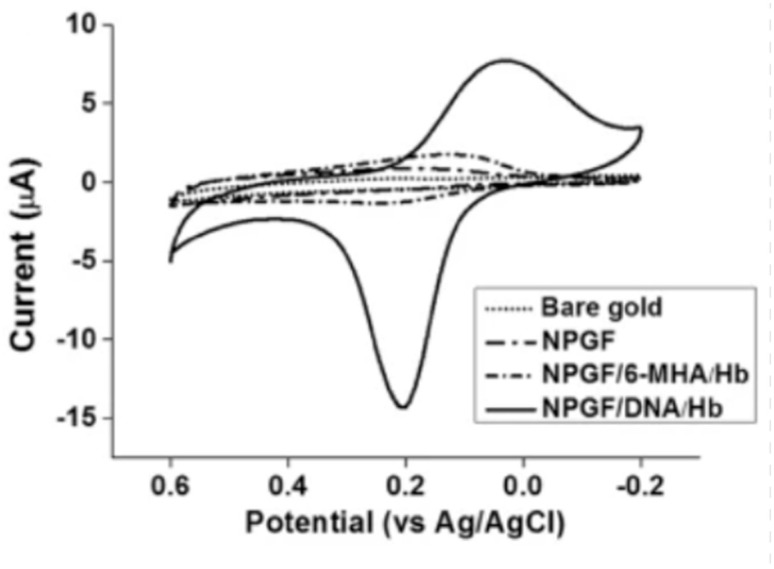
Cyclic voltammograms of bare gold, a nanoporous gold thin film (NPGF) electrode, NPGF/6-MHA/Hb and NPGF/DNA/Hb in PBS [60].

Figure 5 shows the cyclic voltammetry graph of bare gold, nanoporous gold thin film (NPGF), NPGF with hemoglobin immobilized with 6-mercaptohexanoic acid (6-MHA) and NPGF with hemoglobin immobilized with DNA. The bare gold electrode and the NPGF oxidation and reduction peaks prove that with increasing surface area of the electrode, the detection performance improves. 

Several studies have been conducted in an effort to improve the surface area of electrodes by adding various types of nanostructures. Cost efficiency can also be improved by coating low-cost electrodes with high-conductivity materials with nanostructures, for example, gold-nanoparticle-coated, screen-printed carbon electrodes [61]. 

#### 2.2.2. Biorecognition Elements

Biorecognition elements are the sensing materials that are immobilized on the surface of a biosensor platform. Biorecognition material refers to nucleic acid, antibodies, enzymes and cell receptors that are mounted over a transducer and react with the target analyte in the solution to generate a biochemical response [62]. The bioreceptors identify and bind the target while minimizing any interference from other compounds and organisms with high selectivity and sensitivity [63]. The interface between the analyte and the bioreceptor is subsequently translated by the transducer and emits biological signals [64]. Biorecognition elements can be used from naturally occurring states or from synthetic constructs. These elements can be used, along with transducers, before performing electrochemical techniques. 

Common biorecognition elements for virus detection include antibodies and antibody fragments. Antibodies have recognition sites that selectively bind to antigens via a specific region of the antigen known as an epitope [65]. Antibodies can be labelled, or fluorescent or enzymatic tags can be attached. This includes additional reagents and processing steps; however, the binding affinity to the antigen can influence the biosensor’s selectivity [66,67]. Biosensors employing antibody-based biorecognition elements are commonly referred to as immunosensors. Given that antibodies exhibit high selectivity and binding affinity for target species and can be generated for a wide range of infectious agents, they are the gold-standard biorecognition element for virus detection.

Protein detection of COVID-19 can be classified into three components: detection of nucleocapsids, spike proteins and/or a combination of both nucleocapsids and spike proteins [68]. An example of an electrochemical biosensor that detects nucleocapsids is a graphene-based biosensor developed by Alafeef et al. with an integrated electrical readout [69]. This biosensor employs thiol-modified antisense oligonucleotides probes (ssDNA probes) with capped gold nanoparticles (AuNPs) that are specific and selective to detect the nucleocapsid (N-gene) of COVID-19. For detection of spike protein (S protein), an electrochemical sensor with cobalt-functionalized TiO_2_ nanotubes (Co-TNTs) was developed by Vadlamani et al. [70]. The Co-TNTs act as a sensing material to detect the S-protein receptor binding domain of COVID-19. An example of an electrochemical biosensor that detects both nucleocapsids and spike glycoproteins is multiplex rolling circle amplification (RCA) [71]. RCA is an isothermal amplification technique involving DNA or RNA primers annealed to a circular DNA template with the help of DNA or RNA polymerase. An RCA amplicon contains multiple copies of DNA sequences in an end-to-end tandem arrangement known as a concatemer corresponding to a circular template. This type of electrochemical biosensor can rapidly detect S and N genes of COVID-19 in clinical samples, including sandwich hybridization of RCA amplicons with probes and redox active labels known as silica-methylene blue (SiMB) and silica-acridine orange (SiAO). The signal output of the device is detected by differential pulse voltammetry (DPV) [71]. Another example of a COVID-19 sensor using antibodies is a label-free, paper-based electrochemical platform [72]. This type of sensor detects IgM and IgG antibodies that develop in the human body in response to the COVID-19 infection. An immunocomplex is formed between captured antibodies with the immobilized spike protein of COVID-19 antigen on the surface of the electrodes, causing a decrease in current. The current response to the formation of immunocomplex is recorded using the square-wave voltammetry (SWV) method.

Other than antibodies, another type of probe that is gaining traction in the field of biosensors is aptamers. Aptamers are single-stranded oligonucleotides with the ability to bind to various molecules with high selectivity and affinity [73]. Aptamers are isolated from a large random sequence pool through SELEX, which is a process of selection involving systematic evolution of ligands by exponential enrichment [74]. Suitable binding sequences can be isolated from oligonucleotide sequence pools before being amplified for usage; thus, the aptamers can exhibit high selectivity to target species [74]. Aptamers are also known as “chemical antibodies” and show benefits relative to antibodies, such as quick synthesis and excellent stability. Aptamers can also be fabricated at a lower cost than other biorecognition elements, such as antibodies. Aptamers have been used extensively in fundamental research, sensing and even as medications to cure illnesses and stop the spread of viruses [75,76]. 

Pathogen and virus detection using electrochemical biosensors usually employ single-stranded DNA aptamers. Electrochemical aptasensors can be classified into aptasensors without enzymes and aptasensors with enzymes. Aptasensors without enzymes involve the deposition of aptamers on the electrode. Binding between aptamers and the target causes direct changes in impedance. An example of this type of aptasensor is a sensor for detection of avian influenza A (AIV H5N1) [77]. This impedance aptasensor has a select DNA aptamer specific to AIV H5N1 placed on the gold microelectrode surface housed in a microfluidics flow cell, with a detection limit of 0.0128 hemagglutinin units (HAU) and a detection time of 30 min. The DNA aptamer is specific to H5N1 and does not react with other AIV subtypes, such as H1N1, H2N2 or H7N2. 

The next type of electrochemical aptasensor is aptasensors with enzymes, whereby enzyme catalysis aids in the modulation of the electrical signal. An example of this type of aptasensor was developed by Ganabri et al. for the detection of hepatitis C virus (HCV) [78]. The aptamer specific to the HCV core antigen is embedded on graphene quantum dots (GQD) on the surface of a glassy carbon electrode (GCE). This aptasensor has a detection limit of 3.3 pg/mL, with two separate linearity ranges: 10–70 pg/mL and 70–400 pg/mL [78].

Another technique that is rapidly gaining popularity is molecular imprinting technology (MIT) which is an artificial approach to designing strong recognition materials that can resemble natural recognition materials, such as antibodies, and biologically and chemically certify molecules such as amino acids, proteins, nucleotides derivatives, etc. [79,80]. Using MIT, complex linkages between analytes and functional monomers are formed, called molecular imprinted polymers (MIPs), providing a recognition site corresponding to the shape of and aligned to the target molecules [81]. MIPs are porous substances that have been effectively used as synthetic receptors for a wide range of targets, including viruses, biomarkers and explosives. They feature high-affinity binding sites for each target molecule [82]. There are various types of MIP-based sensors; in this review, we focus on examples of electrochemical MIP-based sensors. 

An MIP-based impedimetric sensor was recently developed for the detection of dengue infection in an early stage [83]. For this sensor, non-structural protein 1 (NS1) was used as a template during polymerization on a screen-printed carbon electrode (SPCE) with electrospun polysulfone (PS) nanofibers coated with dopamine. This imprinted sensor was used to measure NS1 in actual human serum samples and achieved satisfactory analytical performance with sensitivities ranging from 95 % to 97.14 % and standard deviations of less than five percent (5%). This sensor can detect NS1 concentrations as low as 0.3 ng/mL. In another study, an MIP-based electrochemical sensor was developed for the detection of COVID-19 [84]. This sensor uses a disposable thin-film metal electrode (Au-TFME) chip modified with MIP film encoded with a selective S1 component of the S protein (nCovS1) [84]. This sensor was evaluated using clinical samples and can detect nCovS1 from samples nasopharyngeal fluid within 15 min. The limit of detection (LOD) value ranges from 15 fM to 64 fM. Table 2 summarizes recent works on the electrochemical detection of COVID-19.

## 3. Point-of-Care Testing for Viruses Using Portable Potentiostats 

Once the major biosensing components, such as the analyte, transducer and biorecognition elements, have been optimized for high sensitivity and selectivity, the next important component that needs to be fine-tuned is the potentiostat. The development of portable potentiostats or usage of commercialized portable potentiostats, such as PalmSens4, for biosensing applications and clinical testing has recently received increasing attention. Over-reliance on RT-PCR tests during the recent COVID-19 pandemic created a considerable testing backlog of due to the necessity of sending the samples to centralized diagnostic laboratories [86]. RT-PCR tests are expensive, resulting in a considerable economic burden, and are not accessible to the majority of the population. In many cases, the time required for sample transportation far exceeds the time spent on testing, further delaying diagnosis [6]. The use of electrochemical sensors coupled with portable potentiostats offers a promising alternative to RT-PCR methods that is more accurate than lateral flow diagnostic kits but not requiring specialized laboratory settings, with the possibility of implementation as a point-of-care device. 

### 3.1. Measurement Techniques 

**Figure 6:**
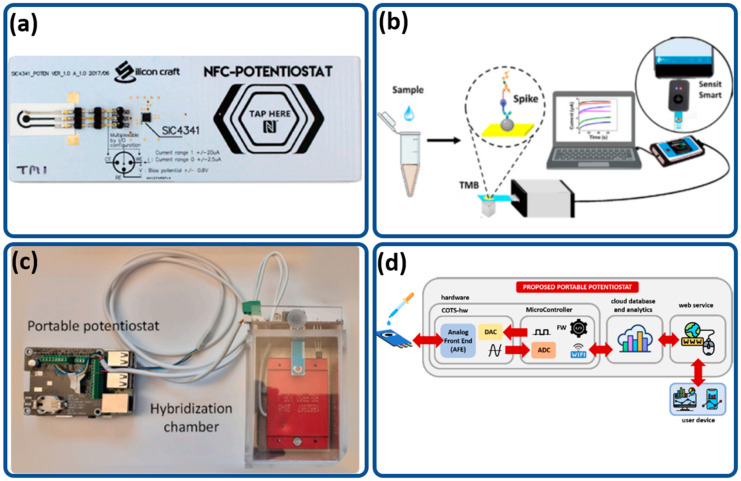
(**a**) SIC4341 circuit board diagram of a portable NFC potentiostat [87]. (**b**) PalmSens4 potentiostat used for SARS-CoV-2 detection [88]. (**c**) Portable potentiostat developed by Kaci et al. (2022) [23]. (**d**) Proposed potentiostat flow developed by Bianchi et al., n.d. [24].

Portable potentiostats can employ various detection techniques, namely amperometry, voltammetry or electrochemical impedance spectroscopy (EIS). Figure 6 shows four portable potentiostats that have been used for virus detection. The credit-card-sized potentiostat shown in Figure 6a uses an SIC4341 chip with embedded near-field communication (NFC) [87]. This potentiostat can run amperometry, voltammetry and EIS techniques and has been used for detection of the hepatitis B virus. Figure 6b shows the scheme of a SARS-CoV-2 virus-sensing system that uses a PalmSens4 potentiostat or Senseit Smart connected to screen-printed sensors. This sensing system uses amperometry to detect angiotensin-converting enzyme host receptor (ACE2) protein by placing anti-spike antibody and anti-ACE2 antibody on the sensing electrodes [88]. The sensitivity of this device is 0.83 mA·mL/mg, and the limit of detection is 22.5 ng/mL of spike protein. The Palmsens4 potentiostat can also be configured for EIS measurements. Palmsens4 has the largest form factor among the reviewed portable potentiostats, but it is equipped with a large battery pack, which improves its portability. As shown in Figure 6b, this potentiostat needs to be connected to a computer for data processing. 

Figure 6c shows an integrated portable potentiostat that uses a Raspberry Pi 3B with an LMP91000 chip for detection of SARS-CoV-2 virus [23]. The use of Raspberry Pi combined with an LMP91000 chip results in a low-cost, compact portable system that can run differential pulse voltammetry (DPV) with a detection limit of 22.1 fM. The system is constructed in such a way as to achieve low cost and high portability. The Raspberry Pi acts as the microcontroller, whereas the LMP91000 is a dedicated chip used to perform electrochemical measurements, such as cyclic voltammetry and DPV. The system also comes with a hybridization chamber that allows for sample preparation. This system has a rapid test time of less than 10 s. Another system shown in Figure 6d that also uses DPV as its measuring technique with an LMP 91000 was developed by Bianchi et al. [24] for detection of hepatitis C virus. This system is equipped with machine learning to improve detection accuracy, with an average accuracy of 98.23%.

### 3.2. Wireless versus Wired Potentiostats 

Wireless potentiostats offer compact, small form factors that are highly portable. Such a compact device can be achieved using near-field communication (NFC) for data communications and readout. An example of a wireless potentiostat is shown in Figure 6a, with an NFC potentiostat developed by Teengam et al. (2021) for detection of hepatitis B virus (HBV) [87]. The use of an NFC allows for signal readout via a smartphone. This small form factor comes at the cost of it being a single-use device, as the sensor is already embedded on the circuit board, with a poor limit of detection (LOD) of 0.17 μg/mL compared to other portable potentiostats, which have LODs in the range of fg/mL. 

**Figure 7:**
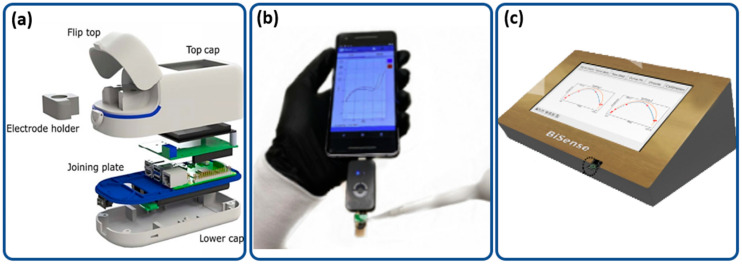
(**a**) Expanded view of the SenSARS portable device [86]. (**b**) PalmSens Sensit module used by Torres et al. (2021) [89]. (**c**) 3D rendering of the Bisense system [90].

Although wired potentiostats are less portable than their wireless counterparts, they often offer more computing power and can perform data analysis. Depending on their complexity, several variations of these potentiostats are available, such as the Palmsense4 [58], the Palmsense Sensit [54], the Bisense [63] and a combination of an LMP 91000 potentiostat chip and a microcontroller [59,60]. These wired potentiostats require either a desktop or laptop computer for data analysis and processing or contain an embedded processor and display unit. Figure 7a shows the SenSARS portable potentiostat system, which is used to detect SARS-CoV-2 virus [86] with an integrated display. Figure 7b shows the PalmSens Sensit module, which has an embedded Emstat potentiostat chip connected to a smartphone [54]. The Sensit module can perform EIS, and the results are graphed on a smartphone display. Although this system is highly portable and does not require an external power supply, experimental settings are constrained due to the limitations of the smartphone app. Figure 7c shows the Bisense system with integrated display [63]. This system is offers multichannel measurements, and although it is slightly larger than the Sensit module, it is still highly portable. 

### 3.3. Microcontroller

Typically, single-chip potentiostats, such as the LMP91000, need to be connected to a microcontroller, such as an Arduino, or a single-board computer, such as Raspberry Pi, for data analysis. The SenSARS system (Figure 7a) uses a Raspberry Pi 4B single-board computer, which has an embedded waveform generation module. Data processing is performed on board by the Raspberry Pi, making it a highly portable standalone system. It also has an integrated display, allowing it to display data directly without the need to connect to an external device. This system has the lowest limit of detection of 1.065 fg/mL amongst all the other potentiostats listed in Table 2. The entire system is lightweight and portable (<200 g), with rapid diagnostic capabilities within 10 min. Although it is not the fastest, is limit of detection is the lowest among the systems reviewed in this article, making it ultra-sensitive. 

The Palmsens Sensit module with an embedded Emstat potentiostat chip and microcontroller is shown in Figure 7b allowcomplex measurement capabilities, such as amperometry, voltammetry and EIS, while maintaining very small form factors and compatibility with smartphones [89]. SARS-CoV-2 detection using this system for 10 mL samples takes just 4 min to complete. The limit of detection is very low, at 2.8 fg/mL, and the system is easily rechargeable via microUSB.

Researchers have also worked on improving measurement throughput, as shown in Figure 7c, which illustrates a multichannel reader known as the Bisense system [63]. This system can handle two simultaneous EIS measurements. Its development is still in progress due to variations in the limit of detection between the two sensors; the LOD of Sensor 1 = 56 fg/mL, and that of Sensor 2 = 68 fg/mL. It has its own embedded display, thus can run as a standalone system. 

In the field of potentiostat research, desirable features include a low limit of detection and a fast execution time. Therefore, it is important to determine the optimal settings that satisfy the optimal conditions with respect to these factors. The capabilities of currently available portable potentiostats are not yet sufficient to detect every virus due to the variation in size and nature of viruses. Table 3 compares currently available portable potentiostats, their limits of detection, detection method and microcontrollers.

## 4. Microfluidic Systems for Electrochemical Measurement Using a Portable Potentiostat

To create a complete point-of-care system for detection of viruses, an electrochemical biosensor is usually incorporated with a microfluidic system. The main advantage of a microfluidic system is that due to its miniature size, it requires only small amounts of reagents and can provide improved sensitivity [91]. Portable microfluidic devices have reduced global cost per analysis and reagent consumption in recent years, resulting in faster analyses due to shorter reactions [92]. Conventional methods of RNA virus detection usually involve serological culture and molecular biology techniques. Integration of these methods into a microfluidic-based device can save a significant amount of time and money. Isothermal PCR methods, such as loop-mediated isothermal amplification (LAMP), are now a viable alternative method for RT-PCR, as they do not require thermal cycling and are easily integrated into portable electrochemical biosensors. LAMP has some fundamental advantages; for example, it can perform amplification at a constant temperature, does not require a thermal cycler, can provide rapid test results and has a high diagnostic capacity, with similar sensitivity and specificity to other methods [22]. RT-PCR tests take 90–120 min per sample set, whereas LAMP tests take only 30 min [93].

**Figure 8:**
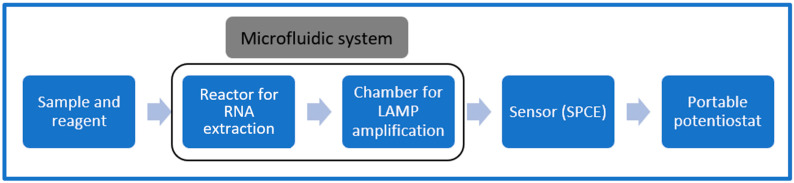
Microfluidic system in a portable electrochemical sensor for virus detection.

Figure 8 shows a typical microfluidic system that uses LAMP for amplification and electrochemistry to detect viruses. The sample and reagents are injected into a microfluidic system, which performs sample mixing for RNA extraction and RT-LAMP reaction to amplify the targeted RNA. The sample is then placed on a screen-printed carbon electrode (SPCE) sensor to measure the presence of virus using a portable potentiostat. The microfluidic system comprises a mixer for RNA extraction and a chamber for LAMP amplification.

Table 4 provides a summary of LAMP methods used in microfluidic devices useful for the detection of RNA viruses. RT-LAMP, unlike commonly used PCR systems, does not require thermal cycles and is carried out at a constant temperature of 60 to 65 degrees Celsius [7]. This simplifies processing of samples, making detection possible with either colorimetric or electrical methods [68,69]. Safavieh et al. developed cellulose-based paper microchips using the RT-LAMP technique to amplify the targeted RNA and detected HIV-1 virus using electrical sensing with LAMP amplicons [69]. They created an RT-LAMP paper microchip assay that could be used to detect HIV-1 in a simple and cost-effective manner. Optical detection can also be achieved using smartphones, making the system easily portable and accessible to areas without centralized laboratories [68,70]. RT-LAMP has also been successfully applied to detect the SARS-CoV-2 virus, as shown in [72]. Successful detection of COVID-19 virus is dependent on the design of the primers that can specifically bind the viral RNA and its fragments on the sensor. With such a design, detection can be easily achieved using either optical or electrical methods. Two other studies have shown that a microfluidic-based RT-LAMP assay can detect the Zika virus and the bacteriophage MS2 virus at a low cost [94].

## 5. Conclusions

Electrochemical biosensors have tremendous potential for point-of-care virus detection due to their portability and simple detection methods. The main limitation of this method is its sensitivity. Numerous studies have been conducted in an attempt to enhance the key elements of electrochemical-based biosensing systems, including transducers, biorecognition methods, analytes, fluidic systems and potentiostats, in order to improve sensing sensitivity and selectivity.

A comparison of the sensitivities demonstrated by electrochemical sensors versus LAM-assisted qPCR and optical sensors (Table 3 and Table 4) shows that electrochemical sensors are the less sensitive option. The detection limit of EIS represents a critical issue, as it does not provide ultra-high resolution. A promising candidate for next-generation virus detection is optical trapping by means of optical tweezers, as they are chip-scaled and can ensure an ultra-high resolution for single [101,102] or multiple viruses [103]. Moreover, integrated photonics offer the possibility of portable systems with a label-free approach to guarantee high resolution within a compact system. MZI [104,105] or high-Q factor resonant cavities [106,107] can be used to ensure a very high detection sensitivity. The aforementioned solutions represent disruptive technologies for in next-generation medicine.

Whereas much work has been done in an attempt to enhance signal output by modifying the surface of transducers and amplifying RNAs, another key aspect that can benefit from further improvement is signal processing units or potentiostats. Currently, commercial devices such as the Palmsens and Bisense offer solutions for handheld mini potentiostats; however, further research on integration with microfluidics and customizable solutions with improved signal accuracies is still needed. More research is also needed on RT-LAMP microfluidics, such they are easily customizable in accordance with the virus of interest for efficient RNA extraction and LAMP amplification. Rapid development and certification of such devices would enable their use as portable screening tools for detection and diagnosis in future pandemics.

## Figures and Tables

**Table 2:** Summary of electrochemical biosensor that are used for the detection of COVID-19 using protein, antibodies, nucleic acid and molecular imprinted polymer.

Author	Instrument	Detection Method	Analysis Time	Limit of Detection	Remarks
Alafeef et al. [69]	Graphene-based electrochemical biosensor (integration of thiol-modified antisense oligonucleotide probes with AuNP caps)	Protein detection: nucleocapsid (N gene) of COVID-19	Less than 5 min	6.9 copies/μL	At present, the device is expected to be integrated with a portable mobile platform that can be used for instantaneous diagnosis of positive COVID-19 cases.
Vadlamani et al. [70]	Cobalt-functionalized TiO_2_ nanotube (Co-TNT)-based electrochemical sensor	Protein detection: spike protein receptor-binding domain (RBD)	~30 s	~0.7 nM levels	This assay has a tendency to be applied for the diagnosis of other recognized respiratory viruses or in conjunction with a suitable metallic element to ensure TNT function.
Chaibun et al. [71]	Multiplex rolling circle amplification (RCA)	Protein detection: nucleocapsid and spike glycoprotein	No data available	1 copy/μL for both N and S genes	The RNA extraction procedure for this assay is currently being optimized, in addition to integration of a smartphone-based biosensor device. Researchers are also working on minimizing the turnaround time for the assay and simplifying the test method.
Yakoh et al. [72]	Label-free, paper-based electrochemical platform	Antibody detection of IgM and IgG	30 min	1 ng/mL	Researchers are working on the direct detection of COVID-19 spike protein of with this assay.
Fan et al. [85]	Entropy-driven amplified electrochemiluminescence (ECL) biosensor	Nucleic acid detection includes the RdRp gene of COVID-19	30 min	As low as 2.67 fM	Not available
Ayankojo et al. [78]	MIP-based electrochemical sensor	S1 component of the S protein (nCovS1)	Not available	4.8 pg/mL	This sensor demonstrates high selectivity to SARS-CoV-2 spike protein compared to other proteins. The sensor is compatible with portable potentiostats and suitable for point-of-care diagnosis.

**Table 3:** Comparison of portable potentiostats used for virus detection.

Reference	Potentiostat/Microcontroller	Brief Description	Detection Method	Size	Voltage Supply	Virus	Limit of Detection (LOD)
[87]	SIC4341	Smartphone-controlled sensor that can operate through an NFC tag sensor. It is unparalleled in terms of portability, with the smallest form factor.	Cyclic voltammetry, amperometry	52 × 18 × 1 mm	1.8V to 3.3V	Hepatitis B Virus	0.17 μg/mL
[88]	PalmSens4	The PalmSens4 potentiostat is used with a sensor linked to anti-ACE2 protein to detect SARS-CoV-2 virus	Amperometry	15.7 × 9.7 × 3.5 cm	5 V	SARS-CoV-2	22.5 ng/mL
[23]	Raspberry Pi 3B LMP91000	Portable point of care (POC) with low cost and high portability designed with a simple user interface for ease of use.	Differential pulse voltammetry	10 × 8 × 4 cm (main module) 12 × 7 × 7 cm (hybridization chamber)	5 V	SARS-CoV-2	22.1 fM
[24]	LMP91000	Portable potentiostat with machine learning, achieving an accuracy of 98.23%.	Differential pulse voltammetry	Not available	5V	Hepatitis C virus	Not specified
[86]	Raspberry Pi 4B	SenSARS: accurate, low cost and portable potentiostat with EIS as the main technique used for viral detection.	Electrochemical impedance spectroscopy	Not specified	5V	SARS-CoV-2	1.065 fg/mL
[89]	Palmsens Sensit	RAPID 1.0 uses Palmsens Sensit as the reader for the sensor with EIS. Other than viral detection, it can also be used to detect bacterial and fungal infection.	Electrochemical impedance spectroscopy	43 × 25 × 11 mm	5 V	SARS-CoV-2	2.8 fg/mL
[90]	Bisense	Bisense is a potentiostat developed to perform EIS on custom-designed dual working electrodes with rapid readings within 1.5 min.	Electrochemical impedance spectroscopy	18 × 15 × 9 cm	Not specified	SARS-CoV-2	56 fg/mL

**Table 4:** Summary of LAMP methods used in microfluidic devices.

Authors	Detection Methods	Detected Virus	Remarks
Song et al. [95]	RT-LAMP and RT-qPCR	Zika virus	High sensitivity at a low cost. Within 40 min, the electricity-free point-of-care diagnostic system detects ZIKV in saliva with a sensitivity of less than 5 PFU of ZIKV per sample.
Ganguli et al. [96]	RT-LAMP and optical sensor	Zika, chikungunya and dengue viruses	Clinically relevant sensitivity. Zika virus detection as low as 1.56 × 10^5^ PFU/mL from whole blood; low reagent consumption. Readout on smartphone.
Safavieh et al. [97]	RT-LAMP and impedance spectroscopy	HIV	Disposable, flexible, low-cost, light, high sensitivity and specificity, rapid amplification, increased stability and low complexity.
Kaarj et al. [98]	RT-LAMP and optical sensor	Zika virus	Limit of detection: 1 copy/μL; simple, quick (15 min) and easily quantifiable with a smartphone.
Lin et al. [99]	RT-LAMP and optical sensor	MS2 virus	Simple to use, low cost, fluorescence intensities 100 times greater than other methods for distinguishing between positive and negative pores.
Huang et al. [100]	RT-LAMP and colorimetric sensor	SARS-CoV-2	Detects SARS-CoV-2 particles in saliva at levels as low as 1.5 copies/µL without the need for RNA isolation.
